# Novel nomograms to predict lymph node metastasis and liver metastasis in patients with early colon carcinoma

**DOI:** 10.1186/s12967-019-1940-1

**Published:** 2019-06-10

**Authors:** Yongcong Yan, Haohan Liu, Kai Mao, Mengyu Zhang, Qianlei Zhou, Wei Yu, Bingchao Shi, Jie Wang, Zhiyu Xiao

**Affiliations:** 1 Guangdong Provincial Key Laboratory of Malignant Tumor Epigenetics and Gene Regulation, Sun Yat-Sen Memorial Hospital, Sun Yat-Sen University, Guangzhou, 510120 China; 2 Department of Hepatobiliary Surgery, Sun Yat-Sen Memorial Hospital, Sun Yat-Sen University, Yanjiang West Road 107#, Guangzhou, 510120 China; 3 RNA Biomedical Institute, Sun Yat-Sen Memorial Hospital, Sun Yat-Sen University, Guangzhou, 510120 China; 4 Department of Gastroenterology and Hepatology, The First Affiliated Hospital, Sun Yat-Sen University, Guangzhou, 510120 China

**Keywords:** Colon carcinoma, Lymph node metastasis, Liver metastasis, Nomogram, Decision curve analysis, Surveillance, epidemiology, and end results

## Abstract

**Background:**

Lymph node status and liver metastasis (LIM) are important in determining the prognosis of early colon carcinoma. We attempted to develop and validate nomograms to predict lymph node metastasis (LNM) and LIM in patients with early colon carcinoma.

**Methods:**

A total of 32,819 patients who underwent surgery for pT1 or pT2 colon carcinoma were enrolled in the study based on their records in the SEER database. Risk factors for LNM and LIM were assessed based on univariate and multivariate binary logistic regression. The C-index and calibration plots were used to evaluate LNM and LIM model discrimination. The predictive accuracy and clinical values of the nomograms were measured by decision curve analysis. The predictive nomograms were further validated in the internal testing set.

**Results:**

The LNM nomogram, consisting of seven features, achieved the same favorable prediction efficacy as the five-feature LIM nomogram. The calibration curves showed perfect agreement between nomogram predictions and actual observations. The decision curves indicated the clinical usefulness of the prediction nomograms. Receiver operating characteristic curves indicated good discrimination in the training set (area under the curve [AUC] = 0.667, 95% CI 0.661–0.673) and the testing set (AUC = 0.658, 95% CI 0.649–0.667) for the LNM nomogram and encouraging performance in the training set (AUC = 0.766, 95% CI 0.760–0.771) and the testing set (AUC = 0.825, 95% CI 0.818–0.832) for the LIM nomogram.

**Conclusion:**

Novel validated nomograms for patients with early colon carcinoma can effectively predict the individualized risk of LNM and LIM, and this predictive power may help doctors formulate suitable individual treatments.

## Background

Colorectal cancer (CRC) is estimated to be the third leading cancer type among new cancer cases and deaths in the United States [1]. In 2018, among the two sexes combined, an estimated 97,220 new cases of colon carcinoma (5.6% of all cancer cases) [2] and an estimated 50,630 (8.3%) deaths from that cause occurred [1]. The poor prognosis and frequent recurrence of colon carcinoma might be related to lymph node metastasis (LNM) and distant metastasis [3]. According to the 7th American Joint Committee on Cancer (AJCC) cancer staging system [4], advanced colon carcinoma (stage III or IV) is diagnosed when LNM or distant metastasis occurs, regardless of the pathologic T (pT) classification. Studies have indicated that 27.3% of patients diagnosed with colon carcinoma develop liver metastasis during the course of their disease, and the proportions of patients with synchronous and metachronous liver metastasis (LIM) were 14.5% and 12.8% [5], respectively. In addition, we found that some advanced colon carcinoma patients remained at pT1 or pT2 due to the migration and invasion capabilities of early colon carcinoma.

When colon carcinoma is detected in a localized stage, the 5-year relative survival is 91.1%. However, the 5-year relative survival of colon carcinoma patients with regional metastasis or distant metastasis were 71.7% and 13.3%, respectively [6]. Therefore, early detection of colon carcinoma metastasis is important for modifying therapeutic strategies and improving patient prognosis.

Most studies of colon cancer metastasis have used lymph nodes to predict the prognosis and recurrence of colon carcinoma [7–11]; research on LIM is much less common. Additionally, there have been few reports or methods to predict LNM and LIM of colon carcinoma. Because the clinicopathological risk factors of LNM and LIM in patients with early colon carcinoma are poorly understood, we attempted to predict the risk factors based on a statistical predictive model.

Nomograms are reliable graphical calculating models that are used to accurately calculate and predict individual risk events by combining all risk factors for tumor development [12, 13]. An increasing number of nomograms are being widely established to provide assistance in formulating individual treatment and follow-up management strategies in several cancers, such as oropharyngeal cancer [14], gastrointestinal stromal tumors [15], adenoid cystic carcinoma [16], bladder cancer [17], and prostate cancer [18]. To the best of our knowledge, no nomograms have been carried out to predict LNM and LIM using data gathered from patients with early colon carcinoma in the Surveillance, Epidemiology, and End Results (SEER) database. Here, we performed nomograms to predict LNM and LIM of early colon carcinoma by combining all relevant risk factors. In addition, decision curve analysis (DCA) and an assessment of clinical impact were conducted to illustrate the clinical utility of the model.

This study aims to evaluate patients with early colon carcinoma using nomograms, discover patients with high risk scores and help to modify therapeutic strategies in clinical application.

## Materials and methods

### Patients and study design

**Fig. 1:**
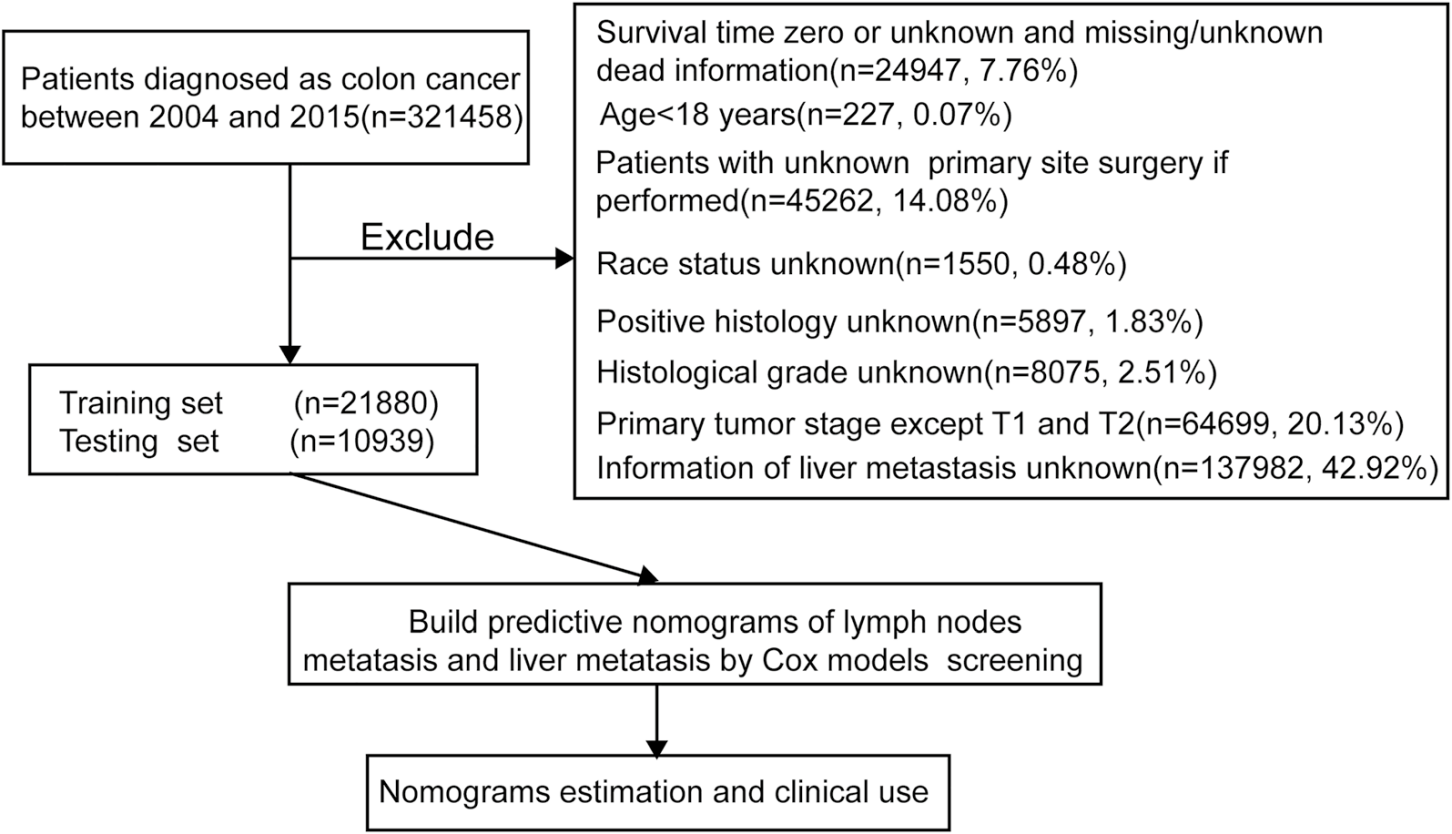
Study flowchart

The records of patients who underwent surgery for pT1 or pT2 colon carcinoma from 2004 to 2015 were retrieved from the SEER 18 registry database using SEER*Stat 8.3.5 software. The flow chart used for data selection is shown in Fig. 1. “The International Classification of Diseases for Oncology (ICD-O-3) Hist/behav, malignant” was used to screen colon carcinoma cases. “Year of diagnosis” ranged from 2004 to 2015. “Derived AJCC Stage Group 7th (2010+)”, “RX Summ-Surg Prim Site (1998+)”, and “Grading and differentiation codes in ICD-O-2” were used in the present study. The codes in Collaborative Stage (CS) (2004+), including tumor size, extension, lymph nodes and metastases, were also collected. The inclusion criteria were as follows: diagnostic confirmation was achieved based on microscopic analysis, and patient background characteristics (age, gender, race and marital status), tumor-related factors [i.e., tumor size and invasion, tumor numbers, histological grade, carcinoembryonic antigen (CEA), LIM, lung metastasis] and survival information were known and available. The exclusion criteria were as follows: death certificate or autopsy only and age < 18 years old. A total of 32,819 cases in the SEER cohort were included and analyzed. We further randomly divided the patients in a 2-to-1 ratio, forming a training set (n = 21,880) for nomogram construction and a validation set (n = 10,939) for internal verification. The data obtained in this study were rooted mainly in the public SEER database, which is available as open-access data. The ethics committee board of Sun Yat-sen Memorial Hospital, Sun Yat-sen University, approved the use of patients with early colon carcinoma for this study.

### Construction and validation of nomograms

Univariable and multivariable analysis were used to identify independent risk factors predictive of LNM and LIM in early colon carcinoma in the SEER discovery set. All variables were screened using the forward stepwise selection method in a multivariate binary logistic regression model [19, 20]. The SEER internal testing set was used to evaluate the predictive reliability and accuracy of the nomograms developed to predict LNM and LIM. For internal validation of the nomogram, we applied a bootstrapping method with 1000 resamples. The predictive performance of the nomograms was measured by a receiver operating characteristic (ROC) curve. Calibration curves were plotted to validate the accuracy and reliability of the nomograms by the Hosmer–Lemeshow test [21].

### Clinical utility

DCA was performed to determine the clinical application value of the nomogram models by calculating the net benefits at each risk threshold probability [22, 23]. The net benefit (NB) was determined by subtracting the proportion of all false-positive patients from the proportion of true positives and weighted by the relative harm caused by forgoing treatment compared with the negative consequences of unnecessary treatment, the NB to the population of using the risk model together with highrisk threshold R is: NB = TPR*P−*(1−R)*FPR*(1−P) (TPR: true-positive rate; FPR: false-positive rate; P: prevalence of the outcome; R: proportion of cases with risk above risk threshold) [24]. Additionally, on the basis of the DCAs, we plotted curves to evaluate the clinical impact of the nomogram to help us more intuitively understand its significant value. These curves display the number of high-risk patients, along with the number of high-risk patients with outcomes of metastasis, at different threshold probabilities in a given population [25].

### Statistical analysis

All statistical analyses were performed using the software IBM SPSS Statistics (version 24, SPSS Inc., Chicago, IL, USA) and the programming language R (version 3.3.4, http://www.R-project.org) for Windows. Patient clinical characteristics are summarized as the mean (s.d.) for continuous measures. The Chi squared test and Student’s t-test were used to compare categorical variables and continuous variables. The ROC curve, nomogram, calibration plots, DCA and clinical impact curves were calculated in R 3.3.4 with relevant packages, such as the survival ROC, rms, calibrate and decision curve packages. The cutoff values of the risk scores from the predictive nomograms of LNM and LIM were determined based on the maximum Youden index of the ROC curve in the training set, and the patients were divided into low- and high-risk groups. All statistical tests were two-sided, and a P value < 0.05 was considered statistically significant.

## Results

### Clinical characteristics of patients

The demographic and clinical characteristics of colon carcinoma patients in both cohorts are summarized in Table 1, and there were no significant differences between the two sets (P>0.05, Table 1). LNM was present in 3111 of 21,880 patients (14.2%) and 30 of 10,939 patients (14.5%) in the training and testing sets, respectively. LIM occurred in 1.5% of patients in the training set and 1.2% of patients in the testing set. There was no statistically significant difference in LNM rate (P = 0.277) or LIM rate (P = 0.06) between the two sets. In the correlation analysis, five variables, namely, histological grade, T classification, tumor size, serum CEA level and overall survival, were significantly correlated (P < 0.001) with LNM (Table 2) and LIM (Table 3) in both the training and testing sets.

**Table 1:** Demographic and clinical characteristics of colon carcinoma patients

Clinicopathological variables	SEER cohort (n = 32,819)			P value
	Entire cohort	Training n = 21,880	Validation n = 10,939	
Age	67.08 (13.40)	67.02 (13.38)	67.19 (13.42)	0.826
Gender				
Female	16,479	10,967	5512	0.659
Male	16,340	10,913	5427	
Marital status				
Married	18,093	12,046	6047	0.922
Single	12,668	8462	4206	
Unknown	2058	1372	686	
Race				
American Indian/Alaska Native	223	154	69	0.735
Asian or Pacific Islander	2467	1650	817	
Black	3956	2658	1298	
White	26,173	17,418	8755	
Histological grade				
Well differentiated	6214	4158	2056	0.172
Moderately differentiated	23,529	15,634	7895	
Poorly differentiated	2615	1761	854	
Undifferentiated	461	327	134	
Histological type				
Adenocarcinoma	28,356	18,917	9439	0.620
Carcinoid tumor	986	644	342	
Neuroendocrine carcinoma	291	194	97	
Mucinous adenocarcinoma	1585	1075	510	
Other	1601	1050	551	
TNM				
I	27,708	18,482	9226	0.408
II	91	58	33	
III	4398	2906	1492	
IV	619	431	188	
T classification				
T1	17,017	11,344	5673	0.990
T2	15,802	10,536	5266	
N classification				
N0	28,114	18,769	9345	0.277
N1	3971	2623	1348	
N2	734	488	246	
M classification				
M0	32,200	21,449	10,751	0.512
M1	619	431	188	
Tumor size				
< 5 cm	24,488	16,294	8194	0.536
≥ 5 cm	3565	2411	1154	
Unknown	4766	3175	1591	
Liver metastasis				
Negative	32,364	21,557	10,807	0.06
Positive	455	323	132	
Lung metastasis				
Negative	32,713	21,801	10,912	0.09
Positive	91	67	24	
Unknown	14	12	2	
Bone metastasis				
Negative	32,793	21,861	10,932	0.227
Positive	14	12	2	
Unknown	11	7	4	
Brain metastasis				
Negative	32,798	21,867	10,931	0.440
Positive	5	4	1	
Unknown	15	9	6	
CEA				
Negative	12,156	8111	4045	0.943
Borderline	80	54	26	
Positive	3385	2270	1115	
Unknown	17,198	11,445	5753	
Tumor number				
1	22,789	15,214	7575	0.898
2	7495	4989	2506	
3	1914	1262	652	
> 3	621	415	206	
Overall survival				
Alive	28,206	18,797	9409	0.812
Dead	4613	3083	1530	

CEA, carcinoembryonic antigen

**Table 2:** Correlations between clinicopathological characteristics of patients and lymph node metastasis in the training and validation sets

Clinicopathological variables	Training set			Validation set		
	Negative	Positive	P value	Negative	Positive	P value
Age	67.36 (13.31)	64.97 (13.58)	*0.023*	67.52 (13.36)	65.18 (13.59)	0.357
Gender						
Female	9421	1546	0.619	4704	808	0.815
Male	9348	1565		4641	786	
Marital status						
Married	10,260	1786	*0.002*	5141	906	*0.017*
Single	7297	1165		3593	613	
Unknown	1212	160		611	75	
Race						
American Indian/Alaska Native	132	22		58	11	*0.001*
Asian or Pacific Islander	1363	287		675	142	
Black	2190	468		1074	224	
White	15,084	2334		7538	1217	
Histologic grade						
Well differentiated	3790	368	*< 0.001*	1885	171	*< 0.001*
Moderately differentiated	13,445	2189		6747	1148	
Poorly differentiated	1292	469		620	234	
Undifferentiated	242	85		93	41	
Histologic type						
Adenocarcinoma	16,276	2641	*< 0.001*	8060	1379	*0.005*
Carcinoid tumor	565	79		312	30	
Neuroendocrine carcinoma	144	50		81	16	
Mucinous adenocarcinoma	896	179		418	92	
Other	888	162		474	77	
T classification						
T1	10,313	1031	*< 0.001*	5125	548	<* 0.001*
T2	8456	2080		4220	1046	
Tumor size						
<5 cm	13,949	2345	<* 0.001*	6984	1210	<* 0.001*
≥ 5 cm	1927	484		919	235	
Unknown	2891	282		1442	149	
CEA						
Negative	6809	1302	<* 0.001*	3405	640	<* 0.001*
Borderline	43	11		22	4	
Positive	1770	500		861	254	
Unknown	10,147	1298		5057	696	
Tumor number						
1	12,996	2218	0.125	6423	1152	*0.035*
2	4315	674		2170	336	
3	1100	162		572	80	
> 3	358	57		180	26	
Overall survival						
Alive	16,258	2539	<* 0.001*	8087	1322	<* 0.001*
Dead	2511	572		1258	272	

Italic values: statistical differences are significant. CEA, carcinoembryonic antigen

**Table 3:** Correlations between clinicopathological characteristics of patients and liver metastasis in the training and validation sets

Clinicopathological variables	Training set			Validation set		
	Negative	Positive	P value	Negative	Positive	P value
Age	67.07 (13.38)	63.70 (12.88)	0.494	67.24 (13.42)	62.78 (13.15)	0.481
Gender						
Female	10,821	146	0.084	5464	48	*0.001*
Male	10,736	177		5343	84	
Marriage						
Married	11,881	165	0.311	5970	77	0.461
Single	8324	138		4156	50	
Unknown	1352	20		681	5	
Race						
American Indian/Alaska Native	152	2	*0.006*	67	2	0.063
Asian or Pacific Islander	1634	16		803	14	
Black	2600	58		1276	22	
White	17,171	247		8661	94	
Histological grade						
Well differentiated	4124	34	<* 0.001*	2043	13	<* 0.001*
Moderately differentiated	15,394	240		7795	100	
Poorly differentiated	1720	41		841	13	
Undifferentiated	319	8		128	6	
Histological type						
Adenocarcinoma	18,632	285	0.067	9327	112	0.154
Carcinoid tumor	641	3		341	1	
Neuroendocrine carcinoma	190	4		94	3	
Mucinous adenocarcinoma	1054	21		501	9	
Other	1040	10		544	7	
T classification						
T1	11,222	122	<* 0.001*	5631	42	<* 0.001*
T2	10,335	201		5176	90	
Tumor size						
<5 cm	16,310	164	<* 0.001*	8120	74	<* 0.001*
≥ 5 cm	2312	99		1118	36	
Unknown	3115	60		1569	22	
CEA						
Negative	8057	54	<* 0.001*	4019	26	<* 0.001*
Borderline	53	1		25	1	
Positive	2097	173		1057	58	
Unknown	11,350	95		5706	47	
Tumor number						
1	15,001	213	0.441	7482	93	0.687
2	4904	85		2474	32	
3	1242	20		646	6	
> 3	410	5		205	1	
Overall survival						
Alive	18,646	151	<* 0.001*	9342	67	<* 0.001*
Dead	2911	172		1465	65	

Italic values: differences are statistically significant

CEA, carcinoembryonic antigen

### Independent significant factors in the training set

To further identify candidate predictors of LNM and LIM, we evaluated all clinicopathological features by binary logistic regression analysis. Risk factors for LNM and LIM were initially identified by univariate logistic regression analysis in the training set (Table 4). Marital status, histological grade, histological type, T classification, tumor size and CEA were associated with LNM. Additionally, there were eight clinicopathological variables related to LIM, namely, age, race, histological grade, histological type, T classification, tumor size, CEA and N classification. A multivariate regression analysis was performed on all factors to verify the risk factors of LNM and LIM (Table 5). Eight variables were actually associated with LNM: age (45–65: odds ratio (OR) 0.83, 95% CI 0.692 to 0.996, P = 0.045; ≥ 65: 0.525, 0.438 to 0.63, P < 0.001), marital status (Single: 0.898, 0.826 to 0.976, P = 0.012; Unknown: 0.806, 0.675 to 0.962, P = 0.017), race (White: 0.732, 0.637 to 0.842, P < 0.001), histological grade (Moderately differentiated: 1.644, 1.442 to 1.875, P < 0.001; Poorly differentiated: 3.641, 3.088 to 4.292, P < 0.001; Undifferentiated: 3.462, 2.609 to 4.593, P < 0.001), histological type (Carcinoid tumor: 1.752, 1.328 to 2.311, P < 0.001; Neuroendocrine carcinoma: 3.74, 2.613 to 5.534, P < 0.001), T classification (T2: 2.221, 2.03 to 2.431, P < 0.001), tumor size (≥ 5 cm: 1.125, 1.003 to 1.262, P = 0.045; Unknown: 0.84, 0.731 to 0.967, P = 0.015) and CEA (Positive: 1.385, 1.228 to 1.561, P < 0.001; Unknown: 0.74, 0.678 to 0.808, P < 0.001). Similarly, LIM was related to five variables: age (≥ 65: 0.532, 0.332 to 0.851, P = 0.008), histologic grade (Moderately differentiated: 1.501, 1.032 to 2.184, P = 0.034; Poorly differentiated: 1.670, 1.028 to 2.714, P = 0.038), tumor size (≥ 5 cm: 2.886, 2.203 to 3.783, P < 0.001; Unknown: 2.463, 1.8 to 3.37, P < 0.001), CEA (positive: 10.436, 7.595 to 14.335, P < 0.001) and N classification (N1: 3.909, 2.999 to 5.095, P < 0.001; N2: 12.131, 8.670 to 16.975, P < 0.001).

**Table 4:** Risk factors for lymph node metastasis and liver metastasis identified by univariate logistic regression analysis

Clinicopathological variables	Lymph node metastasis			Liver metastasis		
	OR	95% CI	P value	OR	95% CI	P value
Age						
<45	1			1		
45–65	0.86	0.729–1.016	0.76	0.774	0.5–1.2	0.253
≥ 65	0.609	0.517–0.717		0.528	0.342–0.814	*0.004*
Gender						
Female	1			1		
Male	1.02	0.946–1.101	0.606	1.222	0.98–1.524	0.075
Marital status						
Married	1			1		
Single	0.917	0.847–0.993	*0.033*	1.194	0.951–1.499	0.128
Unknown	0.758	0.638–0.901	*0.002*	1.065	0.667–1.7	0.791
Race						
Asian or Pacific Islander	1			1		
American Indian/Alaska Native	0.792	0.495–1.265	0.328	1.344	0.306–5.899	0.695
White	0.735	0.642–0.841		1.469	0.884–2.442	0.138
Black	1.015	0.863–1.193	0.858	2.278	1.305–3.976	*0.004*
Histological grade						
Well differentiated	1			1		
Moderately differentiated	1.667	1.493–1.883	<* 0.001*	1.891	1.318–2.713	<* 0.001*
Poorly differentiated	3.739	3.217–4.345	<* 0.001*	2.891	1.829–4.571	<* 0.001*
Undifferentiated	3.617	2.763–4.735	<* 0.001*	3.043	1.396–6.626	*0.005*
Histological type						
Adenocarcinoma	1			1		
Carcinoid tumor	0.862	0.679–1.094	0.222	0.306	0.098–0.957	*0.042*
Neuroendocrine carcinoma	2.14	1.547–2.96	<* 0.001*	1.376	0.508–3.371	0.53
Mucinous adenocarcinoma	1.231	1.043–1.453	*0.014*	1.303	0.833–2.308	0.247
Other	1.124	0.946–1.336	0.183	0.629	0.334–1.185	0.151
T classification						
T1	1			1		
T2	2.461	2.271–2.665	<* 0.001*	1.789	1.426–2.244	<* 0.001*
Tumor size						
<5 cm	1			1		
≥ 5 cm	1.494	1.34–1.666	<* 0.001*	4.212	3.269–5.425	<* 0.001*
Unknown	0.58	0.509–0.66	<* 0.001*	1.894	1.406–2.553	<* 0.001*
CEA						
Negative	1			1		
Borderline	1.338	0.688–2.601	0.391	2.815	0.382–20.726	0.31
Positive	1.477	1.316–1.658	<* 0.001*	12.309	9.035–16.77	<* 0.001*
Unknown	0.669	0.616–0.727		1.249	0.893–1.746	0.194
Tumor number						
1	1			1		
2	0.915	0.834–1.004	0.061	1.222	0.947–1.573	0.123
3	0.863	0.727–1.024	0.091	1.134	0.714–1.8	0.593
>3	0.933	0.703–1.238	0.631	0.859	0.352–2.096	0.738
N classification						
N0	1			1		
N1	NA	NA	NA	4.687	3.64–6.036	<* 0.001*
N2	NA	NA	NA	17.35	12.761–23.59	<* 0.001*

Italic values: differences are statistically significant. OR: odds ratio; 95% CI, 95% confidence interval; CEA, carcinoembryonic antigen; NA, not available

**Table 5:** Risk factors for lymph node metastasis and liver metastasis identified by multivariate logistic regression analysis

Clinicopathological variables	Lymph node metastasis			Liver metastasis		
	OR	95% CI	P value	OR	95% CI	P value
Age						
<45	1			1		
45–65	0.83	0.692–0.996	*0.045*	0.751	0.468–1.206	0.236
≥ 65	0.525	0.438–0.63	<* 0.001*	0.532	0.332–0.851	*0.008*
Marriage						
Married	1					
Single	0.898	0.826–0.976	*0.012*			
Unknown	0.806	0.675–0.962	*0.017*			
Race						
Asian or Pacific Islander	1					
American Indian/Alaska Native	0.759	0.469–1.227	0.261			
White	0.732	0.637–0.842	<* 0.001*			
Black	1.022	0.863–1.21	0.799			
Histological grade						
Well differentiated	1			1		
Moderately differentiated	1.644	1.442–1.875	<* 0.001*	1.501	1.032–2.184	*0.034*
Poorly differentiated	3.641	3.088–4.292	<* 0.001*	1.670	1.028–2.714	*0.038*
Undifferentiated	3.462	2.609–4.593	<* 0.001*	1.939	0.847–4.437	0.117
Histological type						
Adenocarcinoma	1					
Carcinoid tumor	1.752	1.328–2.311	<* 0.001*			
Neuroendocrine carcinoma	3.74	2.613–5.534	<* 0.001*			
Mucinous adenocarcinoma	1.046	0.881–1.241	0.607			
Other	1.118	0.933–1.339	0.226			
T classification						
T1	1					
T2	2.221	2.03–2.431	<* 0.001*			
Tumor size						
<5 cm	1			1		
≥ 5 cm	1.125	1.003–1.262	*0.045*	2.886	2.203–3.783	<* 0.001*
Unknown	0.84	0.731–0.967	*0.015*	2.463	1.8–3.37	<* 0.001*
CEA						
Negative	1			1		
Borderline	1.468	0.743–2.9	0.269	2.763	0.367–20.815	0.324
Positive	1.385	1.228–1.561	<* 0.001*	10.436	7.595–14.335	<* 0.001*
Unknown	0.74	0.678–0.808	<* 0.001*	1.395	0.994–1.958	0.055
N classification						
N0	1			1		
N1	NA	NA	NA	3.909	2.999–5.095	<* 0.001*
N2	NA	NA	NA	12.131	8.670–16.975	<* 0.001*

OR, odds ratio; 95% CI, 95% confidence interval; NA, not available

Italic values: differences are statistically significant

### Development of nomograms for LNM and LIM prediction

**Fig. 2:**
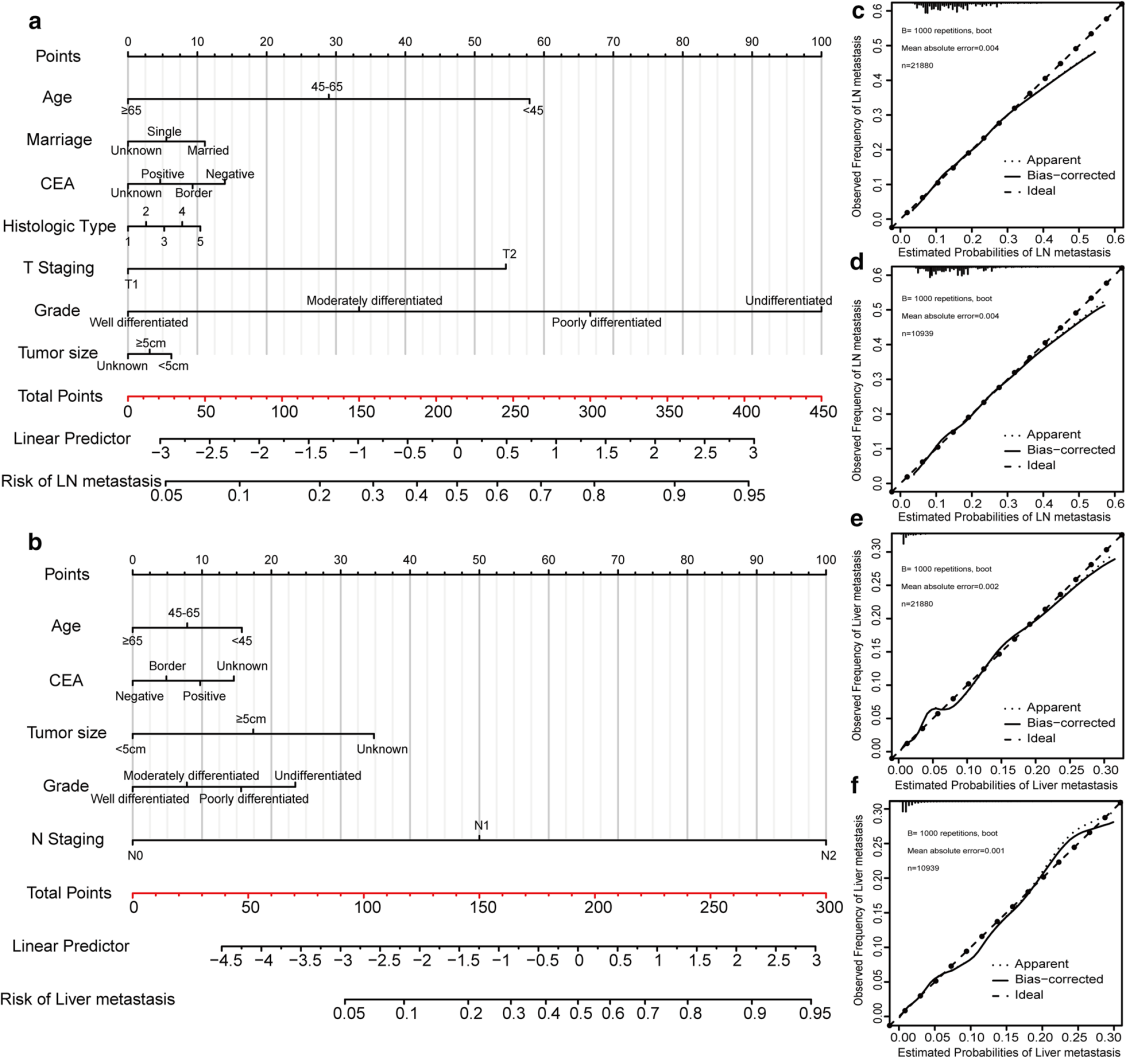
Nomogram and calibration curves for predicting lymph node metastasis and liver metastasis in patients with early colon carcinoma. There are seven factors in the lymph node metastasis prediction nomogram (**a**) and five factors in the liver metastasis prediction nomogram (**b**). Calibration curves for predicting lymph node metastasis and liver metastasis in the training set (**c**, **e**) and in the testing set (**d**, **f**) are shown. All the points assigned on the top point scale for each factor are summed together to generate a total point score. The total point score is projected on the bottom scales to determine the probability of cancer metastasis in an individual. The nomogram-predicted frequency of metastasis is plotted on the x-axis, and the actual observed frequency of metastasis is plotted on the y-axis

Based on the independent risk factors identified in the multivariate regression analysis, two nomograms were developed to predict the possibility of LNM (Fig. 2a) and LIM (Fig. 2b) in patients with early colon carcinoma. Furthermore, point assignments and predictive scores for each variable in the nomogram models were calculated in Table 6. According to the LNM nomogram, histological grade made the largest contribution, followed by T stage, age, marital status, serum CEA level and histological type. N classification made the largest contribution in the LIM nomogram, followed by histological grade, tumor size, serum CEA level and age. The calibration curves for predicting LNM and LIM in the training set (Fig. 2c, e) showed good agreement between predictions and observations.

**Table 6:** Point assignments and predictive scores for each variable in the nomogram models

Variables	Classification	Nomogram score	
		Lymph node metastasis	Liver metastasis
Age	< 45	58	16
	45–65	29	8
	≥ 65	0	0
Marriage	Married	11	NA
	Single	6	NA
	Unknown	0	NA
Histological grade	Well differentiated	0	0
	Moderately differentiated	33	8
	Poorly differentiated	67	16
	Undifferentiated	100	23
Histological type	Adenocarcinoma	0	NA
	Carcinoid tumor	3	NA
	Neuroendocrine carcinoma	5	NA
	Mucinous adenocarcinoma	8	NA
	Other	10	NA
T classification	T1	0	NA
	T2	54	NA
Tumor size	< 5 cm	6	0
	≥ 5 cm	3	17
	Unknown	0	35
CEA	Negative	14	0
	Borderline	9	5
	Positive	5	10
	Unknown	0	15
N classification	N0	NA	0
	N1	NA	50
	N2	NA	100

CEA, carcinoembryonic antigen; NA, not available

### Performance and validation of nomograms for LNM and LIM prediction

**Fig. 3:**
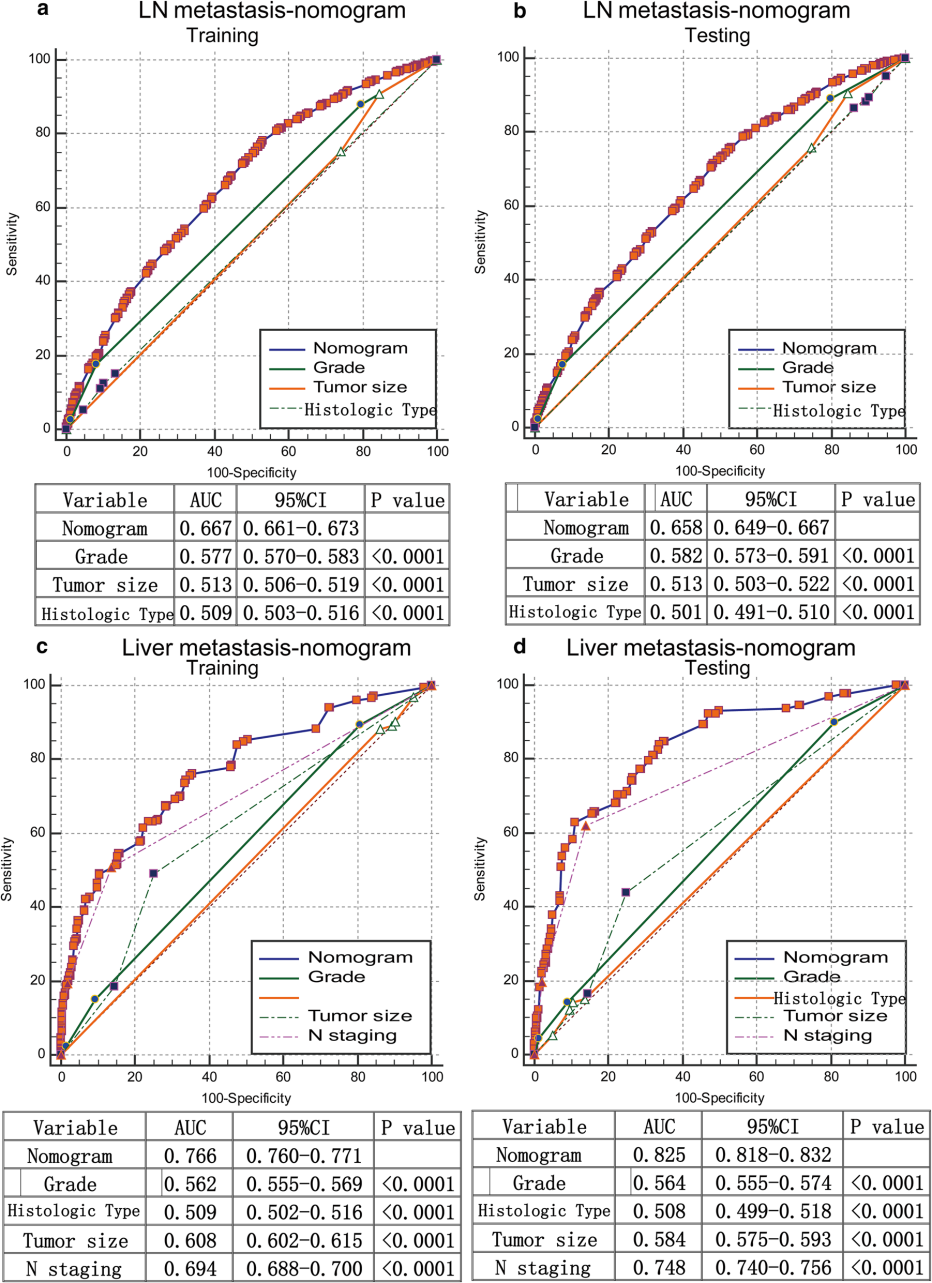
Receiver operating characteristic (ROC) curve analysis for lymph node metastasis and liver metastasis. Comparisons of the predictive values of the nomogram models and clinicopathological risk factors for lymph node metastasis and liver metastasis according to ROC analysis. ROC curves of lymph node metastasis in the training set (**a**) and the testing set (**b**); ROC curves of liver metastasis in the training set (**c**) and the testing set (**d**). The AUC was calculated, and its 95% CI was estimated by bootstrapping. The P values were two-sided. Abbreviations: LN, lymph nodes; ROC, receiver operating characteristic; 95% CI, 95% confidence interval

**Fig. 4:**
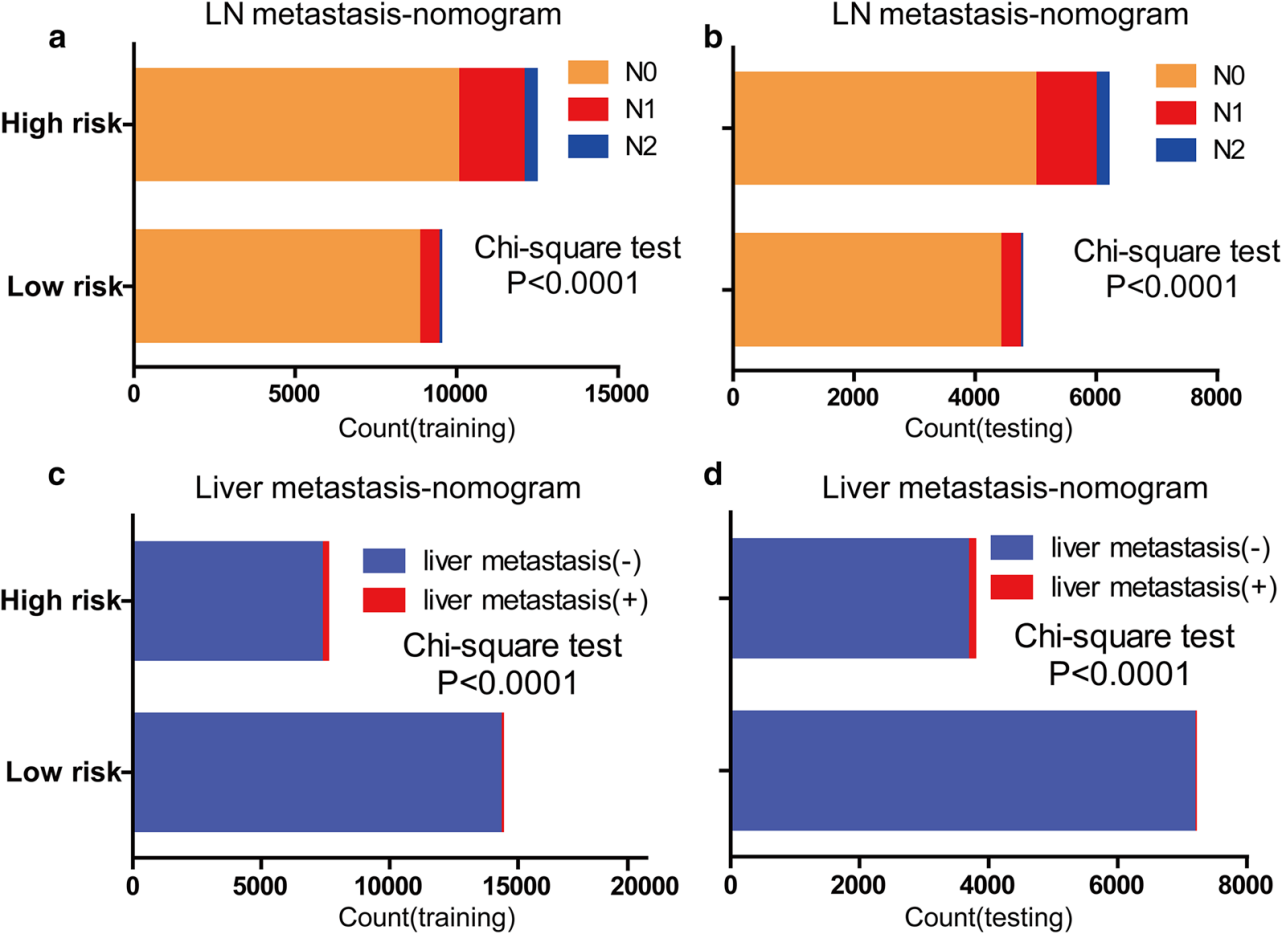
Discriminatory power of the nomograms for lymph node metastasis and liver metastasis, illustrated with bar charts. Risk classification for the predictive nomograms was conducted by the maximum Youden index of the ROC curve, and their performance in distinguishing lymph node metastasis and liver metastasis in the training set (**a**, **c**) and the testing set (**b**, **d**) were plotted

The calibration curves for predicting LNM and LIM demonstrated that the nomograms were generally well calibrated in the testing set (Fig. 2d, f). To compare the predictive values for LNM and LIM of the nomogram models and clinicopathological risk factors, we applied ROC analysis. In the ROC curves of LNM in the training set (Fig. 3a) and the testing set (Fig. 3b), the area-under-the-curve (AUC) values of the nomograms were 0.667 (95% CI 0.661–0.673) and 0.658 (95% CI 0.649–0.667), respectively; these values were significantly larger than the AUCs of grade, tumor size and histological type in both sets (P < 0.0001). Similarly, the AUCs of nomograms of LIM in the training set (Fig. 3c) and the testing set (Fig. 3d), with values of 0.766 (95% CI, 0.760–0.771) and 0.825 (95% CI, 0.818–0.832), respectively, were higher than those for histological grade, histological type, tumor size and N classification. Moreover, we generated bar charts to evaluate the discriminatory power of the nomograms in LNM and LIM after calculating the risk scores from the nomograms. Using the maximum Youden index in the training set, we obtained cutoff values of 79 and 33 for the LNM and LIM nomograms, respectively. All patients were divided into low- and high-risk groups. Patients with predicted high-risk LNM actually had a higher proportion of N1 and N2 classification than the low-risk group in the training set (Fig. 4a). The proportion of N1 and N2 classification in the testing set was near the proportions in the training set (Fig. 4b). Similarly, the high-risk group had a greater possibility of LIM than the low-risk group in both the training and testing sets (Fig. 4c, d).

### Clinical utility

**Fig. 5:**
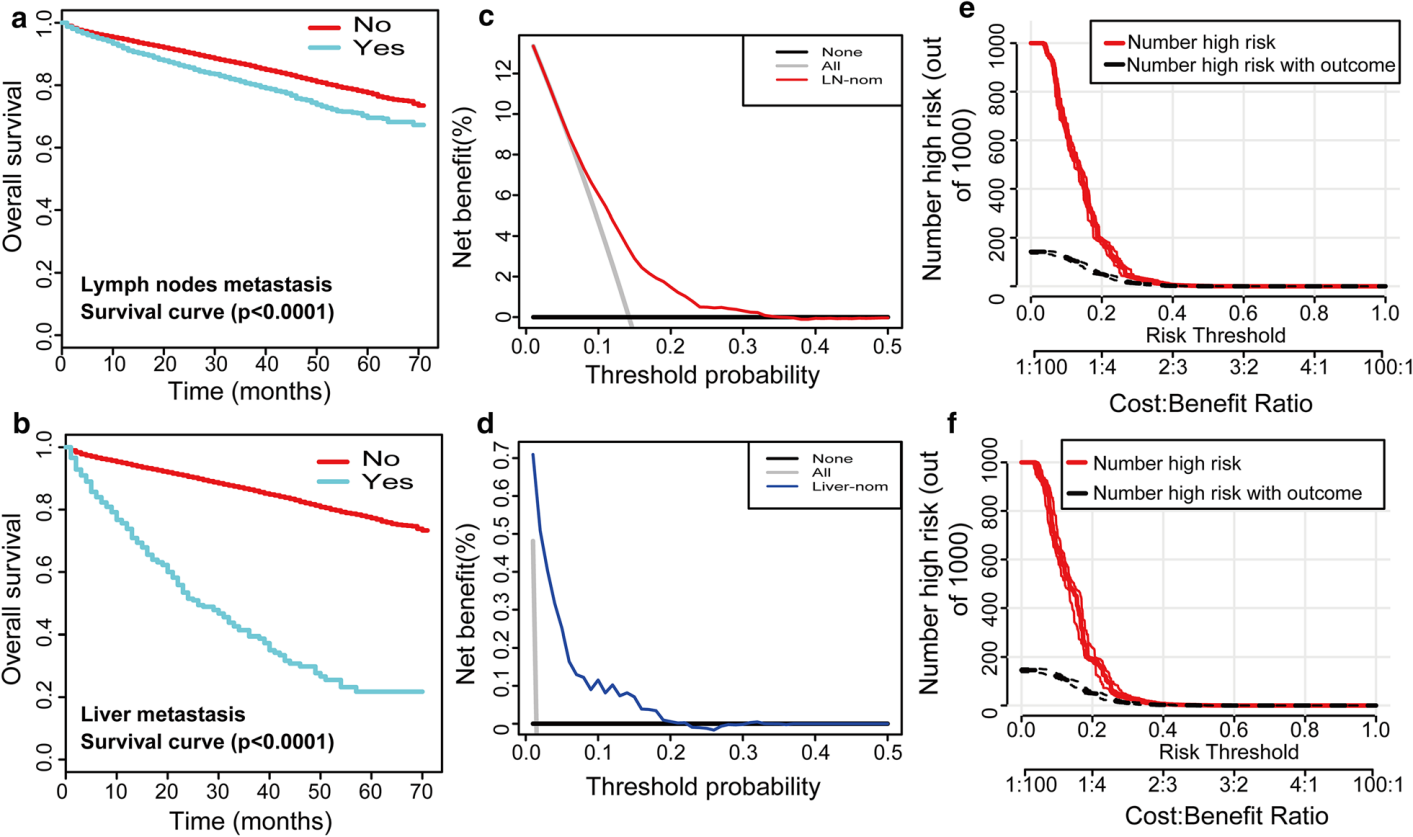
Kaplan–Meier survival curves, decision curve analyses, and clinical impact curves of overall survival for patients. Kaplan–Meier survival curves representing the overall survival of patients with lymph node metastasis (**a**) and liver metastasis (**b**) in the entire SEER cohort. The decision curves of the nomograms for predicting lymph node metastasis (**c**) and liver metastasis (**d**) in the training set were plotted. Clinical impact curves of the nomogram to predict lymph node metastasis in the training set (**e**) and the testing set (**f**) are shown. The y-axis represents the net benefit. The x-axis shows the threshold probability. The horizontal solid black line represents the hypothesis that no patients experienced lymph node metastasis or liver metastasis, and the solid gray line represents the hypothesis that all patients met the endpoint (**c**, **d**). At different threshold probabilities within a given population, the number of high-risk patients and the number of high-risk patients with the outcome were plotted (**e**, **f**)

Kaplan–Meier survival curves of overall survival for patients according to LNM (Fig. 5a) and LIM (Fig. 5b) in the entire SEER cohort verified that patients who were predicted to have LNM or LIM had a significant disadvantage in overall survival (P < 0.0001). DCAs were performed on the nomograms for predicting LNM (Fig. 5c) and LIM (Fig. 5d) in the training set. Threshold probabilities of 0–0.3 for LNM or 0–0.2 for LIM were the most beneficial for predicting LNM and LIM with our nomograms. Based on these DCAs of LNM, we further plotted curves to evaluate the clinical impact of the nomograms to help us more intuitively understand their substantial value. Clinical impact curves of the LNM nomogram in the training set (Fig. 5e) and testing set (Fig. 5f) showed that the model had remarkable predictive power: the predicted number of high-risk patients was always greater than the number of high-risk patients with outcomes of metastasis when the risk threshold was in the range of 0–0.3, and the cost–benefit ratios would be acceptable in the same range.

## Discussion

Colon carcinoma ranks fourth in terms of incidence but fifth in terms of mortality worldwide in 2018. In 2018, among both genders combined, the incidence of colon carcinoma is approximately 1,096,601 new cases, and the mortality is approximately 551,269 [26]. Death from colon carcinoma typically occurs due to distant metastasis, while lymph node metastases are thought to occur before distant metastasis [3]. A study has reported that an increased number of lymph nodes evaluated is associated with increased survival. Therefore, lymph node evaluation is important for the prognosis and treatment of patients with colon cancer and may be a measure of quality care [9]. For distant metastasis, a population-based cancer registry in Burgundy reported that 27.3% of patients diagnosed with colon carcinoma develop LIM during the course of their disease, and the 5-year cumulative metachronous LIM rate was 14.5% in general, 3.7% for TNM stage I tumors, and 13.3% for stage II [5]. Metachronous LIM also contributed greatly to the poor prognosis and recurrence of colon carcinoma.

When metastasis occurs, surgical treatments such as en bloc resections of the affected segments of the bowel and the associated draining lymph nodes [27], as well as adjuvant therapies, should be applied [28]. Partial or total colectomy is performed in the majority of patients with stage I and II colon cancer (84%), while 67% and 40% of patients with stage III and stage IV, respectively, receive chemotherapy in addition to colectomy to lower their risk of recurrence [29]. Several studies have examined the number [9], distribution and size of affected lymph nodes [8] or the ratio of metastatic to examined lymph nodes [7] to evaluate colon cancer survival. Some researchers have focused on mRNA expression of genes related to lymph nodes, such as guanylyl cyclase C (GCC) [11] and metastasis associated in colon cancer 1 (MACC1) [10], to evaluate colon cancer prognosis. It is unknown whether LIM is derived from cancer cells that first colonize intestinal lymph nodes or whether such metastases can form without prior lymph node involvement in colorectal cancer. Enquist et al. found direct hematogenous spread as a dissemination route contributing to CRC liver metastasis in CRC mouse models [30]. Therefore, the correlations between LNM, LIM and tumor recurrence should not be ignored, and in order to modify therapeutic strategies and improve patient prognosis, it is essential to estimate the risks of LNM and LIM in early colon carcinoma. c-MET, a proto-oncogene that initiates a range of signals to regulate various cellular functions, has been suggested to be associated with CRC progression [31] . Hiroya Takeuchi and coworkers reported that c-MET copy numbers in primary CRC of N1/N2-stage patients were significantly higher than the copy numbers in N0 cases (P < 0.03) and that overexpression of c-MET mRNA in primary CRC may be a predictor of tumor invasion and lymph node metastases [32]. Zuo et al. found that serum soluble lectin, which was increased in colon cancer patients with LIM compared to those without metastases, might be a promising new target for intervention in metastasis formation [33]. However, fundamental studies are not a direct way to predict metastasis in daily clinical practice and would be costly even if they could be employed in the clinic. As a result, we focused on clinical studies based on clinicopathological risk factors.

Some researchers have estimated the risk of metastasis using clinicopathological variables and nomograms. A study of 160 patients with early colorectal cancer assessed CT and MRI data to establish imaging criteria for LNM and concluded that a short-diameter size criterion of ≥ 4.1 mm for metastatic lymph nodes showed sensitivity of 78.6% and specificity of 75% [34]. In addition, Yan-qi Huang et al. developed and validated a radiomics-based nomogram incorporating the radiomics signature, CT-imaged lymph node status, and clinical risk factors to facilitate the preoperative individualized prediction of LNM in patients with colorectal cancer [24]. Martin R. Weiser and colleagues developed a colon cancer recurrence nomogram to predict relapse based on the number of positive and negative lymph nodes, lymphovascular invasion and other risk factors [35]. Because nomograms are commonly used tools for prognosis in oncology and medicine [22] and straight scales are useful for relatively simple calculations, we decided to build a nomogram for LNM and LIM prediction in early colon carcinoma. The scarcity of studies examining liver metastasis in colon carcinoma supported our decision to develop a nomogram for predicting LIM in early colon carcinoma.

Two nomograms were constructed and validated for predicting LNM and LIM in patients with early colon carcinoma. The nomogram for LNM incorporates seven factors, namely, age, marital status, CEA, histological type, T classification, histological grade and tumor size, while the nomogram for LIM includes five factors: age, CEA, tumor size, histological grade and N classification.

Both of the nomograms demonstrated good agreement between predictions and observations in the training and testing sets. Furthermore, better diagnostic efficiencies were shown by ROC curves in comparison with histologic grade, histologic type, tumor size and N classification. In particular, the AUCs of the LIM nomograms were calculated with values of 0.766 (0.760–0.771) and 0.825 (0.818–0.832), respectively, in the training set and the testing set.

However, the nomograms might not be useful with greater AUCs and good agreement between predictions and observations [13]. Therefore, decision curve analyses were performed in the present study. DCA is a novel method for evaluating diagnostic tests, prediction models and molecular markers. This method can also be easily extended to many of the applications common to performance measures for prediction models [22]. Here, good clinical utility was indicated in the proper range. Moreover, the clinical impact of the LNM nomogram on the basis of DCA, Kaplan–Meier survival curves and bar charts with Chi squared tests was used to improve the discriminatory power of the nomograms. The nomograms for predicting LNM and LIM actually possess good prediction efficiencies as judged by the methods above.

In our study, a large number of cases in the SEER dataset were chosen and randomly divided into a training set and an internal testing set. Our purpose was to evaluate the prediction of LNM and LIM in early colon carcinoma from large quantities of patient data, which are convincing and readily available in clinical decision making. For clinical application, it is important to make the assessment of risk factors as convenient as possible. We considered the variables needed in our nomogram to be prevalent in clinical practice and convenient to acquire. The limitations of our study are the lack of external validation for the nomogram and the absence of genetic markers. Because the testing set in this study was derived from the same SEER dataset as the training study, potentially leading to overfitting of the model, external validation at our hospital or another institution should be performed. Multicenter validation with a large sample size is preferable because it yields high-level evidence for clinical application. In addition, our research did not incorporate genetic markers because clinical risk factors are easier to collect. However, a combination of clinical variables and genetic markers may improve the prediction of LNM and LIM in patients with early colon carcinoma.

## Conclusions

In conclusion, based on the clinical risk factors identified in a large population-based cohort, we established the first practical nomograms that can objectively and accurately predict individualized risk of LNM and LIM. Moreover, the internal cohort validation results demonstrate that the two nomograms perform well and have high accuracy and reliability. Our nomograms were demonstrated to be clinically useful in DCAs, and they should therefore help clinicians to improve individual treatment, make clinical decisions and guide follow-up management strategies for patients with early colon carcinoma.

## Data Availability

Please contact the corresponding author for all data requests.

## Abbreviations

LIM: liver metastasis
LNM: lymph node metastasis
AUC: area under the curve
SEER: surveillance, epidemiology, and end results
DCA: decision curve analysis
ROC: receiver operating characteristic

## References

[1] Siegel RL, Miller KD, Jemal A (2018). Cancer statistics, 2018. CA Cancer J Clin.

[2] Benson AB, Venook AP, Al-Hawary MM, Cederquist L, Chen YJ, Ciombor KK, Cohen S, Cooper HS, Deming D, Engstrom PF (2018). NCCN Guidelines insights: colon cancer, Version 2. 2018. J Natl Compr Canc Netw.

[3] Ulintz PJ, Greenson JK, Wu R, Fearon ER, Hardiman KM (2018). Lymph node metastases in colon cancer are polyclonal. Clin Cancer Res.

[4] Edge SB, American Joint Committee on Cancer, American Cancer Society (2010). AJCC cancer staging handbook : from the AJCC cancer staging manual.

[5] Manfredi S, Lepage C, Hatem C, Coatmeur O, Faivre J, Bouvier AM (2006). Epidemiology and management of liver metastases from colorectal cancer. Ann Surg.

[6] Siegel RL, Miller KD, Fedewa SA, Ahnen DJ, Meester RGS, Barzi A, Jemal A (2017). Colorectal cancer statistics, 2017. CA Cancer J Clin.

[7] Berger AC, Sigurdson ER, LeVoyer T, Hanlon A, Mayer RJ, Macdonald JS, Catalano PJ, Haller DG (2005). Colon cancer survival is associated with decreasing ratio of metastatic to examined lymph nodes. J Clin Oncol.

[8] Yamaoka Y, Kinugasa Y, Shiomi A, Yamaguchi T, Kagawa H, Yamakawa Y, Furutani A, Manabe S (2017). The distribution of lymph node metastases and their size in colon cancer. Langenbecks Arch Surg.

[9] Chang GJ, Rodriguez-Bigas MA, Skibber JM, Moyer VA (2007). Lymph node evaluation and survival after curative resection of colon cancer: systematic review. J Natl Cancer Inst.

[10] Isella C, Mellano A, Galimi F, Petti C, Capussotti L, De Simone M, Bertotti A, Medico E, Muratore A (2013). MACC1 mRNA levels predict cancer recurrence after resection of colorectal cancer liver metastases. Ann Surg.

[11] Sargent DJ, Shi Q, Gill S, Louvet C, Everson RB, Kellner U, Clancy TE, Pipas JM, Resnick MB, Meyers MO (2014). Molecular testing for lymph node metastases as a determinant of colon cancer recurrence: results from a retrospective multicenter study. Clin Cancer Res.

[12] Kluth LA, Black PC, Bochner BH, Catto J, Lerner SP, Stenzl A, Sylvester R, Vickers AJ, Xylinas E, Shariat SF (2015). Prognostic and prediction tools in bladder cancer: a comprehensive review of the literature. Eur Urol.

[13] Balachandran VP, Gonen M, Smith JJ, DeMatteo RP (2015). Nomograms in oncology: more than meets the eye. Lancet Oncol.

[14] Fakhry C, Zhang Q, Nguyen-Tan PF, Rosenthal DI, Weber RS, Lambert L, Trotti AM, Barrett WL, Thorstad WL, Jones CU (2017). Development and validation of nomograms predictive of overall and progression-free survival in patients with oropharyngeal cancer. J Clin Oncol.

[15] Lee CK, Goldstein D, Gibbs E, Joensuu H, Zalcberg J, Verweij J, Casali PG, Maki RG, Cioffi A, McArthur G (2015). Development and validation of prognostic nomograms for metastatic gastrointestinal stromal tumour treated with imatinib. Eur J Cancer.

[16] Ganly I, Amit M, Kou L, Palmer FL, Migliacci J, Katabi N, Yu C, Kattan MW, Binenbaum Y, Sharma K (2015). Nomograms for predicting survival and recurrence in patients with adenoid cystic carcinoma. An international collaborative study. Eur J Cancer.

[17] Mitra AP, Lam LL, Ghadessi M, Erho N, Vergara IA, Alshalalfa M, Buerki C, Haddad Z, Sierocinski T, Triche TJ (2014). Discovery and validation of novel expression signature for postcystectomy recurrence in high-risk bladder cancer. J Natl Cancer Inst.

[18] Gravis G, Boher JM, Fizazi K, Joly F, Priou F, Marino P, Latorzeff I, Delva R, Krakowski I, Laguerre B (2015). Prognostic factors for survival in noncastrate metastatic prostate cancer: validation of the glass model and development of a novel simplified prognostic model. Eur Urol.

[19] Sauerbrei W, Boulesteix AL, Binder H (2011). Stability investigations of multivariable regression models derived from low- and high-dimensional data. J Biopharm Stat.

[20] Collins GS, Reitsma JB, Altman DG, Moons KG (2015). Transparent reporting of a multivariable prediction model for individual prognosis or diagnosis (TRIPOD): the TRIPOD statement. BMJ.

[21] Kramer AA, Zimmerman JE (2007). Assessing the calibration of mortality benchmarks in critical care: the Hosmer–Lemeshow test revisited. Crit Care Med.

[22] Vickers AJ, Cronin AM, Elkin EB, Gonen M (2008). Extensions to decision curve analysis, a novel method for evaluating diagnostic tests, prediction models and molecular markers. BMC Med Inform Decis Mak.

[23] Vickers AJ, Elkin EB (2006). Decision curve analysis: a novel method for evaluating prediction models. Med Decis Making.

[24] Huang YQ, Liang CH, He L, Tian J, Liang CS, Chen X, Ma ZL, Liu ZY (2016). Development and validation of a radiomics nomogram for preoperative prediction of lymph node metastasis in colorectal cancer. J Clin Oncol.

[25] Kerr KF, Brown MD, Zhu K, Janes H (2016). Assessing the clinical impact of risk prediction models with decision curves: guidance for correct interpretation and appropriate use. J Clin Oncol.

[26] Bray F, Ferlay J, Soerjomataram I, Siegel RL, Torre LA, Jemal A (2018). Global cancer statistics 2018: GLOBOCAN estimates of incidence and mortality worldwide for 36 cancers in 185 countries. CA Cancer J Clin.

[27] Nelson H, Petrelli N, Carlin A, Couture J, Fleshman J, Guillem J, Miedema B, Ota D, Sargent D (2001). National Cancer institute expert P: guidelines 2000 for colon and rectal cancer surgery. J Natl Cancer Inst.

[28] Benson AB, Bekaii-Saab T, Chan E, Chen YJ, Choti MA, Cooper HS, Engstrom PF, Enzinger PC, Fakih MG, Fenton MJ (2013). Metastatic colon cancer, version 3.2013: featured updates to the NCCN guidelines. J Natl Compr Canc Netw.

[29] Miller KD, Siegel RL, Lin CC, Mariotto AB, Kramer JL, Rowland JH, Stein KD, Alteri R, Jemal A (2016). Cancer treatment and survivorship statistics, 2016. CA Cancer J Clin.

[30] Enquist IB, Good Z, Jubb AM, Fuh G, Wang X, Junttila MR, Jackson EL, Leong KG (2014). Lymph node-independent liver metastasis in a model of metastatic colorectal cancer. Nat Commun.

[31] Trusolino L, Comoglio PM (2002). Scatter-factor and semaphorin receptors: cell signalling for invasive growth. Nat Rev Cancer.

[32] Takeuchi H, Bilchik A, Saha S, Turner R, Wiese D, Tanaka M, Kuo C, Wang HJ, Hoon DS (2003). c-MET expression level in primary colon cancer: a predictor of tumor invasion and lymph node metastases. Clin Cancer Res.

[33] Zuo Y, Ren S, Wang M, Liu B, Yang J, Kuai X, Lin C, Zhao D, Tang L, He F (2013). Novel roles of liver sinusoidal endothelial cell lectin in colon carcinoma cell adhesion, migration and in-vivo metastasis to the liver. Gut.

[34] Choi J, Oh SN, Yeo DM, Kang WK, Jung CK, Kim SW, Park MY (2015). Computed tomography and magnetic resonance imaging evaluation of lymph node metastasis in early colorectal cancer. World J Gastroenterol.

[35] Weiser MR, Landmann RG, Kattan MW, Gonen M, Shia J, Chou J, Paty PB, Guillem JG, Temple LK, Schrag D (2008). Individualized prediction of colon cancer recurrence using a nomogram. J Clin Oncol.

